# 

**Published:** 1891-10

**Authors:** 


CONTENTS:

PAGE

ORIGINAL ARTICLES:
- Rachitis. By W. L. Williams, V. S. .... 477
- The Transatlantic Cattle Trade and its Regulations from a Veterinary Point of View. By Williamson Bryden, V. S. .... 495

SOCIETY PROCEEDINGS:
- United States Veterinary Medical Association .... 502
  - President's Address .... 502
  - Comitia Minora—Minutes .... 504
  - Members Elected .... 506
  - Members Dropped .... 510
  - Committee Reports: ....
    - Intelligence and Education. Austin Peters .... 514
    - Army Legislation .... 527
    - Publication. W. H. Hoskins .... 528
    - National and International Meat Inspection. W. S. Williams .... 529
    - Secretary's Report .... 544
    - Special College Committee .... 547
  - Discussion Reports .... 548
  - Election of Officers .... 562
  - Applications for Membership .... 567
  - Papers: ....
    - Barren Mares. C. C. Lyford .... 570
    - Identification of Animals. R. S. Huidekoper .... 570
  - Discussion of Papers .... 571

REVIEW DEPARTMENT:
- Books and Pamphlets Received .... 586

